# Extracellular vesicles produced by bone marrow mesenchymal stem cells attenuate renal fibrosis, in part by inhibiting the RhoA/ROCK pathway, in a UUO rat model

**DOI:** 10.1186/s13287-020-01767-8

**Published:** 2020-06-26

**Authors:** Zhengzhou Shi, Qi Wang, Youbo Zhang, Dapeng Jiang

**Affiliations:** 1 Department of Urology, Shanghai Children’s Medical Center, Shanghai Jiao Tong University School of Medicine, 1678 Dongfang Road, Shanghai, 200127 China; 2 Department of Pediatric Surgery, Nantong Maternal and Child Health Hospital, Nantong, Jiangsu China

**Keywords:** UUO, Renal interstitial fibrosis, Apoptosis, Oxidative stress, MFG-E8, RhoA/ROCK

## Abstract

**Background:**

Extracellular vesicles produced by bone marrow mesenchymal stem cells (BMSC-EVs) can play important roles in the repair of injured tissues. Though numerous studies have reported the effect of EVs on renal fibrosis, the underlying mechanisms remain unclear. We hypothesized that BMSC-EVs containing milk fat globule–epidermal growth factor–factor 8 (MFG-E8) could attenuate renal fibrosis by inhibiting the RhoA/ROCK pathway.

**Methods:**

We investigated whether BMSC-EVs have anti-fibrotic effects in a rat model of renal fibrosis, in which rats were subjected to unilateral ureteral obstruction (UUO), as well as in cultured HK2 cells. Extracellular vesicles from BMSCs were collected and co-cultured with HK2 cells during transforming growth factor-β1 (TGF-β1) treatment. HK2 cells co-cultured with TGF-β1 were also treated with the ROCK inhibitor, Y-27632.

**Results:**

Compared with the Sham group, UUO rats displayed fibrotic abnormalities, accompanied by an increased expression of α-smooth muscle actin and Fibronectin and reduced expression of E-cadherin. These molecular and pathological changes suggested increased inflammation in damaged kidneys. Oxidative stress, as evidenced by an increased level of MDA and decreased levels of SOD1 and Catalase, was also observed in UUO kidneys. Additionally, activation of cleaved caspase-3 and PARP1 and increased apoptosis in the proximal tubules confirmed tubular cell apoptosis in the UUO group. All of these phenotypes exhibited by UUO rats were suppressed by treatment with BMSC-EVs. However, the protective effect of BMSC-EVs was completely abolished by the inhibition of MFG-E8. Consistent with the in vivo results, treatment with BMSC-EVs reduced inflammation, oxidative stress, apoptosis, and fibrosis in HK-2 cells stimulated with TGF-β1 in vitro. Interestingly, treatment with Y-27632 protected HK-2 cells against inflammation and fibrosis, although oxidative stress and apoptosis were unchanged.

**Conclusions:**

Our results show that BMSC-EVs containing MFG-E8 attenuate renal fibrosis in a rat model of renal fibrosis, partly through RhoA/ROCK pathway inhibition.

## Background

Chronic kidney disease (CKD), and its major pathological feature renal fibrosis, is an emerging health issue worldwide [1, 2]. Patients with CKD experience progressive deterioration of renal function that can progress to end-stage renal failure. Though considerable effort has been dedicated to finding ways to ameliorate renal fibrosis in these patients, few specific therapeutic strategies are available that effectively delay or prevent the progression of renal tubulointerstitial fibrosis (TIF) to end-stage renal failure [3, 4]. Bone marrow mesenchymal stem cells (BMSCs), a multipotent progenitor cell type capable of repair, regeneration, and immunomodulation, are widely seen as a promising therapeutic option for renal disease [5]. For example, one trial found that activated MSCs can repair injured tissue by secreting a variety of anti-inflammatory factors [6]. BMSCs are generally recognized for their ability to self-renew and differentiate into multiple lineages.

Recently, it has become increasingly clear that BMSC-derived extracellular vesicles (BMSC-EVs) have important biological functions and molecular mechanisms and may serve as an alternative to therapies based on BMSC transplantation [7, 8]. The lower immunogenicity and simple storage process make BMSC-EVs more clinically promising than their producing cells [8]. However, the biological role of BMSC-EV-regulated organ damage and the mechanism underlying this process remain elusive, so more detailed research is needed to determine the molecular functions of BMSC-EVs in this setting.

Milk fat globule–epidermal growth factor–factor 8 (MFG-E8), also known as lactadherin, is a secreted multifunctional glycoprotein commonly found in human milk fat globules [9, 10]. MFG-E8 has aroused widespread interest over the last decade for its role in mediating various biological processes and pathophysiological functions, including the apoptotic clearance of dead cells in cardiovascular disease and the attenuation of oxidative stress in the amelioration of early brain injury [11, 12]. There is also growing evidence that MFG-E8 regulates the RhoA/ROCK pathway. Kudo et al. [13] found that MFG-E8 inhibits the stimulatory effect of allergic inflammation on RhoA activity, thereby reducing the contraction of airway smooth muscle in humans and mice. Additionally, MFG-E8 regulates gastrointestinal motility by preventing RhoA activation through a phosphatase and tensin homolog-dependent mechanism [14], while Su et al. [15] demonstrated an anti-fibrotic function for MFG-E8 as one of the components in secretomes derived from MSCs. Thus, we postulated that BMSC-EVs attenuate renal fibrosis by delivering MFG-E8, which then regulates the RhoA/ROCK signaling pathway.

To test our hypothesis that BMSC-EVs protect against inflammation, mitochondrial dysfunction, oxidative stress, and apoptosis, thereby decreasing renal fibrosis, we delivered BMSC-EVs to a rat model of unilateral ureteral obstruction (UUO). Our findings provide important insights into BMSC-EVs as a potential treatment option for CKD and clarify the mechanisms underlying their effect on renal fibrosis.

## Methods

### Chemicals and reagents

The primary antibodies used in our study, anti-α-SMA (ab5694), anti-Fibronectin (ab2413), anti-CD34 (ab81289), anti-hepatocyte growth factor (HGF; ab83760), anti-ED1 (ab31630), and anti-CD44 (ab189524), were obtained from Abcam (Cambridge, UK). Anti-E-cadherin (20874-1-AP), anti-SOD1 (10269-1-AP), anti-Catalase (21260-1-AP), anti-cleaved caspase-3 (19677-1-AP), anti-Decorin (14667-1-AP), and anti-PARP1 (13371-1-AP) were from Proteintech (Wuhan, China). Anti-RhoA (sc-418), anti-ROCK1 (sc-17794), anti-Col1α1 (sc-293182), and anti-MFG-E8 (sc-271574) were from Santa Cruz Technology (Santa Cruz, CA, USA). Anti-p-MYPT1 (4563T) was from Cell Signaling Technology (Danvers, MA, USA). Anti-CD11b (201807) and anti-CD90 (206105) were from BioLegend (San Diego, CA, USA). Anti-CD34 (A0378) and anti-pigment epithelium-derived factor (PEDF; A3475) were from ABclonal (Woburn, MA, USA). Glyceraldehyde 3-phosphate dehydrogenase (GAPDH; AG019), the enhanced ATP assay kit (S0027), JC-1 (C2006), and the reactive oxygen species assay kit (S0033) were from Beyotime (Shanghai, China). Y-27632 (S104921) was from Selleck (Houston, TX, USA). Recombinant human TGF-β1 (100-21) was from Peprotech (Rocky Hill, NJ, USA). Mesenchymal stem cell adipogenic differentiation medium (RASMX-90031), mesenchymal stem cell chondrogenic differentiation medium (RASMX-90041), and mesenchymal stem cell osteogenic differentiation medium (RASTA-90021) were from Cyagen (Santa Clara, CA, USA). The RhoA activation assay combo biochem kit (BK030) was from Cytoskeleton (Denver, CO, USA). PKH26 (PKH26GL) and PKH67 (PKH67GL) were from Sigma (St Louis, MO, USA). Recombinant MFGE8 (Q08431) was from R&D systems (Minneapolis, MN, USA). Kit for the determination of MDA (A003-1) was from Jiancheng (Nanjing, China). Fetal bovine serum (16000044) was from Gibco (CA, USA).

### Animals

Eight-week-old male Sprague-Dawley (SD) rats (weighing 180–220 g) were used for this study. The animal experiments were approved by the Ethics Committee of Laboratory Animal Science, Shanghai Children’s Medical Center, Shanghai Jiao Tong University School of Medicine (SCMCIACUC-K2019025). All rats were housed in a specific pathogen-free animal room under standard conditions with free access to water and food.

### Animal experiments

Seventy-eight (78) male SD rats were randomly divided into 13 groups (*n* = 6 in each group): Sham group, UUO group (day 7), UUO group (day 14), Sham + BMSC-EVs group, UUO + BMSC-EVs group (0.05 mg/kg), UUO + BMSC-EVs group (0.5 mg/kg), UUO + BMSC-EVs group (1 mg/kg), UUO + BMSC-EVs group (postoperative day 1), UUO + BMSC-EVs group (postoperative day 3), UUO + BMSC-EVs group (postoperative day 7), UUO + BMSC-EVs^Ctrl^, UUO + BMSC-EVs^shMFGE8^, and UUO + rMFGE8. Briefly, rats were anesthetized with 60 mg/kg pentobarbital and kept on a heated surface to maintain a body temperature of approximately 36.5 °C. In the UUO group, the left ureter was exposed via abdominal incision and then ligated to the ureteral–pelvic junction with 5-0 silk sutures. In the Sham group, the left ureter was physically exposed but not ligated. A solution containing different concentrations of EVs (0.05, 0.5, and 1 mg/kg EVs in 200 μl phosphate-buffered saline [PBS]) was injected into rats on postoperative day 1 in the UUO + EVs group, and a dose of 0.5 mg/kg EVs in 200 μl PBS was injected into rats from the Sham + EVs group. To test differences between treatment protocols (times), rats received injections of 0.5 mg/kg EVs on postoperative day 1, 3, or 7. Rats in the Sham and UUO groups received the same volume of PBS intravenously. For the UUO + rMFGE8 group, 0.02 mg/kg rMFGE8 was administered intravenously. All rats were sacrificed by injecting a pentobarbital overdose on postoperative day 7 or 14, and the kidneys were collected for further investigation.

### BMSC isolation and culturing

BMSCs were isolated from the bone marrow of 4-week-old SD rats. The cells were cultured in Dulbecco’s modified Eagle’s medium (DMEM) supplemented with 10% fetal bovine serum (FBS) and 1% penicillin–streptomycin in a 37 °C incubator with 5% CO_2_. The marrow was plated in T75 culture flasks, and nonadherent hematopoietic cells were removed with PBS 3 days later, followed by the addition of fresh culture medium; the medium was subsequently changed every 2–3 days. When the BMSCs reached 80–90% confluence, they were digested with 0.25% trypsin. The cells were passaged three times prior to verification of their identity. Flow cytometry was used to screen the cells for CD44, CD90, CD11b, and CD34 expression. The cells were also tested for their ability to differentiate into adipocytes, osteocytes, and chondrocytes. BMSCs at passages 3–5 were used for further experiments after the characterization of BMSCs.

### Lentiviral vector construction and transduction

To downregulate MFG-E8 expression in BMSC-EVs, BMSCs were divided into two groups: BMSCs transfected with null lentivirus vector (BMSCs^Ctrl^) and BMSCs transfected with a lentiviral vector (final concentration, 100 nM) designed to knock down MFG-E8 expression (BMSCs^shMFGE8^). Lipofectamine 2000 was used to perform the transfections. The medium was removed 24 h after transfection and replaced with fresh medium containing 10% FBS. BMSC-EVs were purified from BMSCs^Ctrl^ or BMSCs^shMFGE8^ as described below.

### Isolation and identification of BMSC-EVs

BMSC-EVs were isolated and identified as the manufacturer’s instructions. Briefly, BMSCs at 80–90% confluency were rinsed with PBS and cultured for 48 h in Mesen Gro MSC medium. The conditioned medium was then collected and centrifuged at 300×*g* for 10 min followed by another round of centrifugation at 2000×*g* for 10 min at 4 °C to remove cellular debris and dead cells. The supernatant was then ultracentrifuged at 100,000×*g* for 70 min at 4 °C to obtain a pellet containing EVs, which was rinsed in 200 μl PBS and ultracentrifuged again at 100,000×*g* for another 70 min at 4 °C. The protein content of the BMSC-EVs was quantitated using a bicinchoninic acid (BCA) protein assay kit. Western blot and transmission electron microscopy were used to examine the quality and morphology of the BMSC-EVs, and qNano was used to assess their size. EVs were labeled with fluorescent dye PKH26 or PKH67 for in vitro and in vivo tracing [16]. The purified BMSC-EVs were stored at − 80 °C for later experiments.

### HK-2 cell culturing and treatment

Human renal proximal tubular epithelial (HK-2) cells (XY Biotechnology, Guangzhou, China) were cultured in DMEM/F12 with 10% FBS supplemented with 100 U/ml penicillin and 100 μg/ml streptomycin. Cells were maintained at 37 °C in a 5% CO_2_/95% air atmosphere with nearly 100% relative humidity. The cells were incubated with TGF-β1 at a final concentration of 10 ng/ml for 72 h with or without 30 μg/ml BMSC-EVs in the presence of TGF-β1. For the TGF-β1 + rMFGE8 group, the cells were incubated with rMFGE8 at 100 ng/ml. To inhibit ROCK, Y-27632 (10 μM) was added to cells treated with TGF-β1 for the final 24 h of incubation.

### Renal morphology analysis

For histological examination, the kidneys were isolated, fixed in 4% formaldehyde overnight, and embedded in paraffin. Hematoxylin and eosin (HE) staining was performed according to the manufacturer’s instructions. Renal interstitial lesions were characterized by the degree of changes to the glomerulus and tubules. At least 10 randomly chosen non-overlapping fields of view at a magnification of × 200 were observed and recorded for each section. The histopathological features of the kidney were assessed as previously described [17]. Renal interstitial lesions were characterized by the degree of changes to the glomerulus and tubules and were graded on a scale from 0 to 4: 0, normal; 1, changes to < 25% of the cortex; 2, changes to 25–50% of the cortex; 3, changes to 50–75% of the cortex; and 4, changes to > 75% of the cortex. Masson’s trichrome staining was used to assess the degree of renal interstitial fibrosis based on the amount of collagen deposition observed at × 200 magnification. An optical microscope equipped with image analysis software was applied to analyze images of the renal interstitium. The total area occupied by fibrotic lesions was calculated for arbitrarily chosen fields of view and expressed as the percentage of fibrotic area relative to the entire image.

### Renal immunofluorescence staining

Immunofluorescence staining on renal paraffin sections was performed as described previously [18]. For CD34 and Col1α1 staining, paraffin-embedded sections were used. After dewaxing, CD34 and Col1α1 antigens were retrieved by heating the slides in citrate buffer for 10 min. Kidney sections were blocked with 1% bovine serum albumin for 30 min, followed by incubation with primary antibodies overnight. Secondary antibody incubation to stain nuclei was conducted for 1 h. The slides were visualized using a fluorescence microscope.

### Renal immunohistochemical analysis

For IHC staining, paraffin-embedded kidney sections were rehydrated and incubated in 10 mM SOD1ium citrate with 0.05% Tween for antigen retrieval [18]. After blocking, the sections were incubated with anti-α-SMA (ab5694), anti-Fibronectin (ab2413), anti-ED1 (ab31630), and anti-E-cadherin (20874-1-AP) at 4 °C overnight, followed by incubation with a horseradish peroxidase–conjugated secondary antibody for 1 h at 37 °C. All images were recorded using Image-Pro Plus 6.0.

### RhoA activation assay

The RhoA activation assay was performed using a GTP pulldown assay kit according to the manufacturer’s instructions. After cells were lysed, 300–500 μg of total protein was added to 10 μl RBD-binding beads, then incubated at 4 °C on a rotator for 1 h, washed three times with PBS, and resuspended in Laemmli buffer. Finally, the protein samples were heated at 100 °C for 10 min and stored at − 20 °C.

### Western blot

Total protein was extracted by kidney samples or HK-2 cells in ice-cold radioimmunoprecipitation assay buffer containing a protease inhibitor cocktail, followed by homogenization and subsequent centrifugation at 12000×*g* for 20 min. Pellets were then discarded and the protein concentration was quantified using the BCA assay kit. Next, 4 × volume of loading buffer was added to each supernatant for Western blot. Equal amounts of each protein sample (40 μg/lane) were loaded onto an 8–12% sodium dodecyl sulfate–polyacrylamide gel, which was electrophoresed for 2 h and then transferred to a polyvinylidene fluoride membrane. The membrane was blocked for 1.5 h at room temperature with 5% nonfat milk followed by incubation at 4 °C overnight with primary antibodies against the following proteins: α-SMA (1:500), E-cadherin (1:1000), Fibronectin (1:1000), Catalase (1:500), SOD1 (1:500), cleaved caspase-3 (1:1000), PARP1 (1:1000), MFG-E8 (1:1000), HGF (1:1000), PEDF (1:1000), Decorin (1:1000), and GAPDH (1:1000). The membrane was then incubated with a horseradish peroxidase–conjugated goat anti-mouse secondary antibody or with rabbit IgG for 1 h at room temperature and then washed three times. Protein bands were detected by enhanced chemiluminescence and imaged. Semi-quantitative analysis was performed by measuring band intensities for three experiments using ImageJ software.

### Quantitative real-time PCR

Total RNA was isolated from kidney tissues using TRIzol according to the manufacturer’s instructions. Reverse transcription was carried out to synthesize complementary DNA (cDNA) from 500 ng total RNA using 2 μl 5× primescript RT Master Mix (TAKARA) in a 10-μl reaction volume. Quantitative real-time PCR was performed using the qPCR Master Mix (TAKARA), primers (designed and synthesized by Shanghai Sangon Biological Co., Ltd., Shanghai, China), and RNase-free ddH_2_O in a 10-μl reaction volume. An Applied Biosystems® 7500 Fast Real-time PCR System was used to run the following program: 95 °C for 30 s, then 40 cycles of 95 °C for 5 s and 60 °C for 34 s, followed by 95 °C for 15 s, 60 °C for 1 min, and 95 °C for 15 s. The threshold cycle (Ct) was recorded by the instrument’s software, and fold changes in mRNA expression were calculated according to the comparative Ct method (2^-△△Ct^). Results were normalized to mRNA expression in the kidney tissues of rats from the Sham group. Rat primer sequences are listed in Table 1.

**Table 1:** Rat primers used for qRT-PCR analysis

Gene	Primer sequence
α-SMA	F: 5′-CAGGGAGTGATGGTTGGAAT-3′ R: 5′-GGTGATGATGCCGTGTTCTA-3′
E-cadherin	F: 5′-CTCAGTGTTTGCTCGGCGTTTGC-3′ R: 5′-GCTCTGGGTTGGATTCAGAG-3′
Fibronectin	F: 5′-GATTCTTCTGGCGTCTGCAC-3′ R: 5′-GCCCCGGAACATGAGGATAG-3′
IL-1β	F: 5′-AGCAGCTTTCGACAGTGAGG-3′ R: 5′-CTCCACGGGCAAGACATAGG-3′
IL-6	F: 5′-AGAAAAGAGAGTTGTGCAATGGCA-3′ R: 5′-GGCAAATTTCCTGGTTATATCC-3′
TNF-α	F: 5′-CAACCAGGCCATCAGCAACAACAT-3′ R: 5′-TCTGTGGGTTGTTCACCTCGAACT-3′
IL-10	F: 5′-GGACTTTAAGGGTTACTTGGG-3′ R: 5′-AGAAATCGATGACAGCGTCG-3′
GAPDH	F: 5′-TGACTCTACCCACGGCAAGTTCAA-3′ R: 5′-ACGACATACTCAGCACCAGCATCA-3′

### Measurement of oxidative stress

A portion of the kidney tissues was homogenized in saline solution for analysis of the MDA level. To evaluate the degree of lipid peroxidation, MDA content was measured using commercial reagent kit according to the manufacturer’s instructions. Oxidative stress in kidney tissues was also determined by Western blot analysis of SOD1 and Catalase expression at day 14. Oxidative stress in HK-2 cells was assessed using the ROS detection kit, as previously described [19]. Cells were cultured with DCFH-DA for 20 min at 37 °C, washed three times with PBS, then ROS levels observed using a fluorescent microscope.

### Analysis of apoptosis

Apoptosis was assessed using three different methods. First, apoptosis in kidney tissues was determined by Western blot analysis of cleaved caspase-3 and PARP1 expression. Second, a terminal deoxynucleotidyl transferase dUTP nick end labeling (TUNEL) assay was performed using an in situ cell death detection kit according to the manufacturer’s instructions (Roche Applied Science, Indianapolis, IN) to examine apoptosis in kidney tissues. The number of apoptotic cells was counted under a fluorescent microscope at × 200 magnification. Third, HK-2 cell apoptosis was detected by flow cytometry using an annexin V-fluorescein isothiocyanate (FITC)/propidium iodide (PI) apoptosis detection kit. Briefly, cells were collected and resuspended at 1 × 10^6^/300 μl of binding buffer and incubated for 5 min with 10 μl PI solution and 5 μl annexin V-FITC solution. A total of 10,000 events were collected, and the proportion of apoptotic cells was calculated.

### Measurement of mitochondrial membrane potential (MMP)

The mitochondrial membrane potential assay kit with JC-1 was used according to the manufacturer’s recommendations. J-aggregates showed red fluorescence in cells with a high ΔΨm, while the JC-1 monomer showed green fluorescence in cells with a low ΔΨm. Cultured cells were treated as required for a specific time, and then stained with freshly prepared JC-1 for 20 min at 37 °C in the dark. Next, the supernatant was removed, and cells were washed twice with JC-1 staining buffer (1×). After replacing medium with fresh culture medium, the cells were observed and photographed by fluorescence microscopy, and the ratio of green to red fluorescence was calculated.

### ATP content assay

The enhanced ATP assay kit was used to measure the ATP content in kidney tissues or HK2 cells after injury according to the manufacturer’s protocol. In brief, dissected kidney tissue (approximately 20 mg) was added to 0.2 ml of lysis buffer from the kit and homogenized in a 1-ml all-glass Dounce tissue grinder (Kimble Chase Life Science, Vineland, NJ). The culture medium of HK2 cells seeded in 6-well plates was discarded, and 0.2 ml of lysis buffer was added to each well. The solution containing dissociated tissue cells or cultured cells was centrifuged at 12000×*g* for 5 min at 4 °C for subsequent determination. ATP detection buffer was added to supernatants (100 μl/well), and then 20-μl samples of ATP standards were added to 96-well plates for detection by a luminometer. The values of ATP were assessed by a standard curve as described in the kit.

### Statistical analysis

Data are presented as the mean ± standard deviation (SD). Significant differences were examined by one-way analysis of variance with Bonferroni correction; *P* < 0.05 was deemed to be statistically significant. All statistical analyses were performed using SPSS version 20.0 statistical software.

## Results

### Characterization of BMSCs and BMSC-EVs

**Fig. 1:**
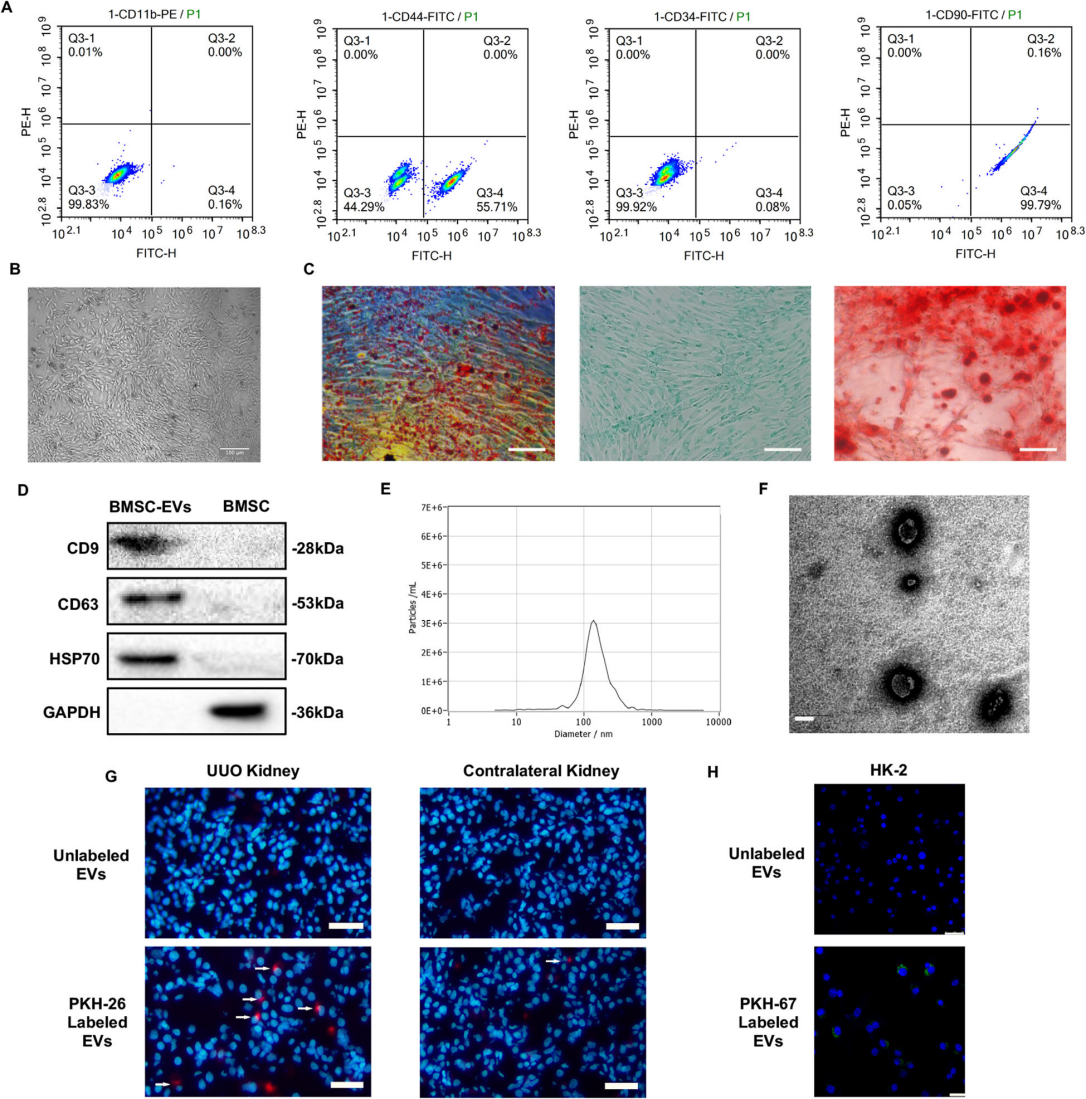
Characterization of BMSCs and BMSC-derived extracellular vesicles. **a** Flow cytometric analysis for detection of BMSC surface markers. **b** Morphology of BMSCs. Scale bar 100 μm. **c** Adipogenic, chondrogenic, and osteogenic differentiation potentials of BMSCs. Scale bar 100 μm. **d** Western blot was used to confirm the expression of EV-specific markers CD9, CD63, and HSP70. **e** Particle size distribution. **f** Representative transmission electron microscopy image of BMSC-EVs, showing a typical spheroid shape. Scale bar 100 nm. **g** Localization of PKH26 fluorescence in the UUO kidney and contralateral kidney. The control group was injected with unlabeled EVs. Scale bar 25 μm. **h** Localization of PKH67 fluorescence in HK-2 cells was determined by fluorescent microscopy. Scale bar 25 μm

The cells were long and spindle-shaped (Fig. 1b), which is highly consistent with previous reports [20]. Flow cytometry analysis showed that these cells exhibited high levels of the characteristic markers CD90 and CD44 (99.79% and 55.71% respectively) and very low levels of CD34 and CD11b (0.08% and 0.01% respectively) (Fig. 1a). Third-generation cells were successfully differentiated into adipogenic, chondrogenic, and osteogenic cells (Fig. 1c). These results indicate that the BMSCs were successfully isolated and cultured. Isolated BMSC-EVs exhibited typical cup-shaped morphology with a diameter of approximately 30–1000 nm when observed by transmission electron microscopy (Fig. 1e, f) and expressed high levels of the characteristic marker proteins CD9, CD63, and HSP70 by Western blot (Fig. 1d). Tracked EVs shown in vitro and in vivo (Fig. 1g, h), which indicated that there were many more labeled EVs on the UUO side than on the contralateral side in vivo.

### Treatment with BMSC-EVs reduces pathological changes and renal fibrosis in UUO rats

**Fig. 2:**
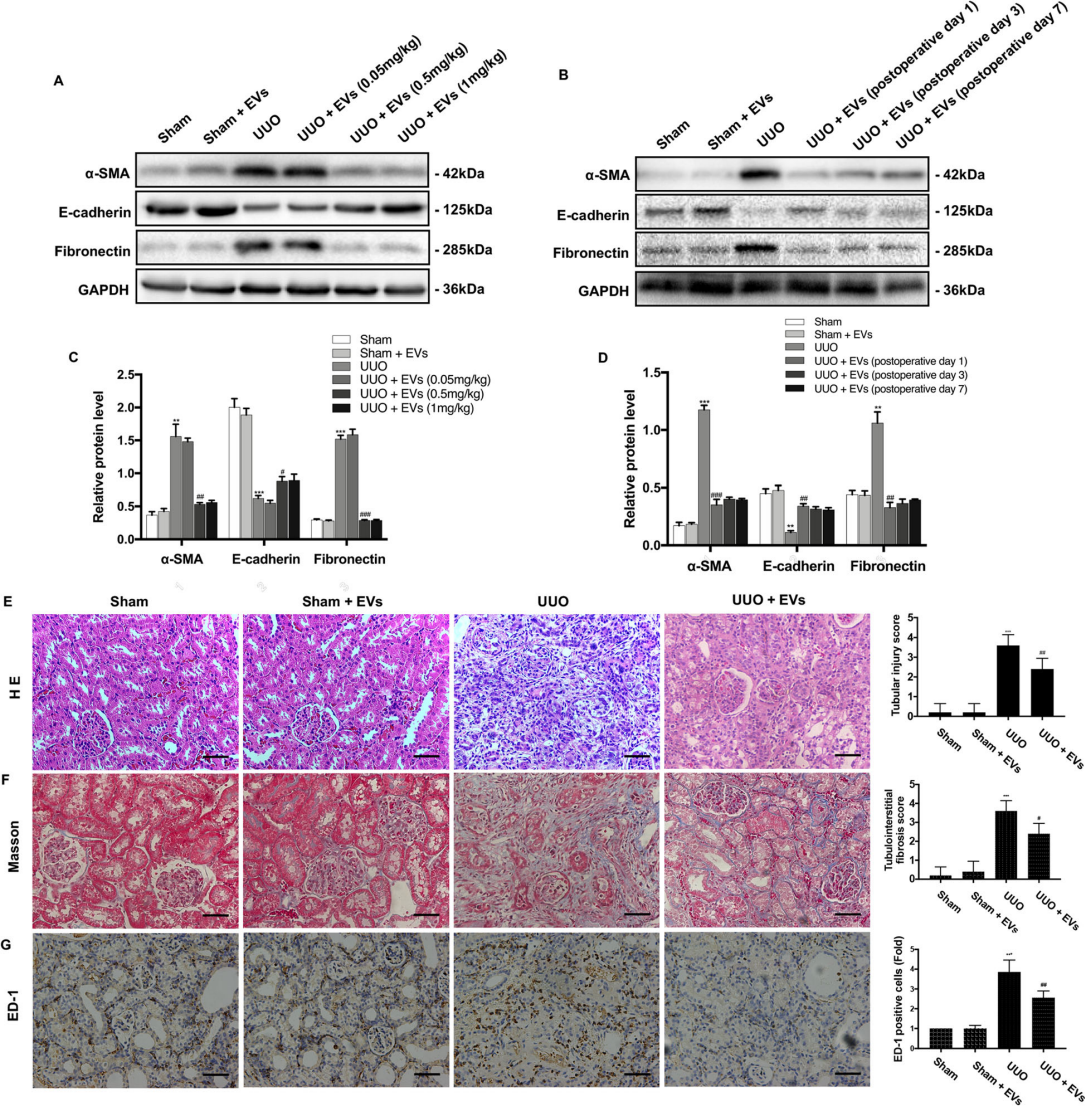
Effects of BMSC-EVs on the tubular damage and collagen deposition in UUO kidneys. **a** BMSC-EVs protect against renal fibrosis in a dose-dependent manner. The expressions of α-SMA, E-cadherin, and Fibronectin were determined by Western blot. **b** BMSC-EVs protect against renal fibrosis at different treatment times. **c** The protein levels of α-SMA, E-cadherin, and Fibronectin were expressed as arbitrary densitometric units and normalized by the value of GAPDH. **d** The protein levels of α-SMA, E-cadherin, and Fibronectin were expressed as arbitrary densitometric units and normalized by the value of GAPDH. **e** Representative image of the HE staining from kidneys of Sham, Sham + BMSC-EVs, UUO, and UUO + BMSC-EVs rats. The bar graph depicts tubular injury scores based on HE staining. Scale bar 100 μm. **f** Representative image of Masson’s trichrome staining from kidneys of Sham, Sham + BMSC-EVs, UUO, and UUO + BMSC-EVs rats. The bar graph depicts renal interstitial fibrosis scores based on Masson’s trichrome staining. Scale bar 100 μm. **g** Representative images illustrating the infiltration of ED-1-positive macrophages of rat kidneys in different groups. Scale bar 100 μm. Quantification of the number of ED-1-postive macrophages pre-field in different groups. Scale bar 150 μm. **P < 0.01, ***P < 0.001 (compared with the Sham group); ^#^*P* < 0.05, ^##^*P* < 0.01, ^###^*P* < 0.001 (compared with the UUO group)

To investigate whether the intravenous administration of BMSC-EVs protects against renal fibrosis in UUO rats, we assessed the role of BMSC-EVs using different doses and treat times. Notably, the UUO + BMSC-EVs group exhibited no difference compared with the UUO group after treatment with BMSC-EVs at 0.05 mg/kg. Protein expression levels of α-SMA and Fibronectin were markedly decreased by BMSC-EVs at 0.5 mg/kg and 1 mg/kg, with no significant difference between these two groups (Fig. 2a, c). Therefore, BMSC-EVs at 0.5 mg/kg in 200 μl PBS were chosen for following studies. We observed no differences between the different treatment times of BMSC-EV administration (Fig. 2b, d).

**Fig. 3:**
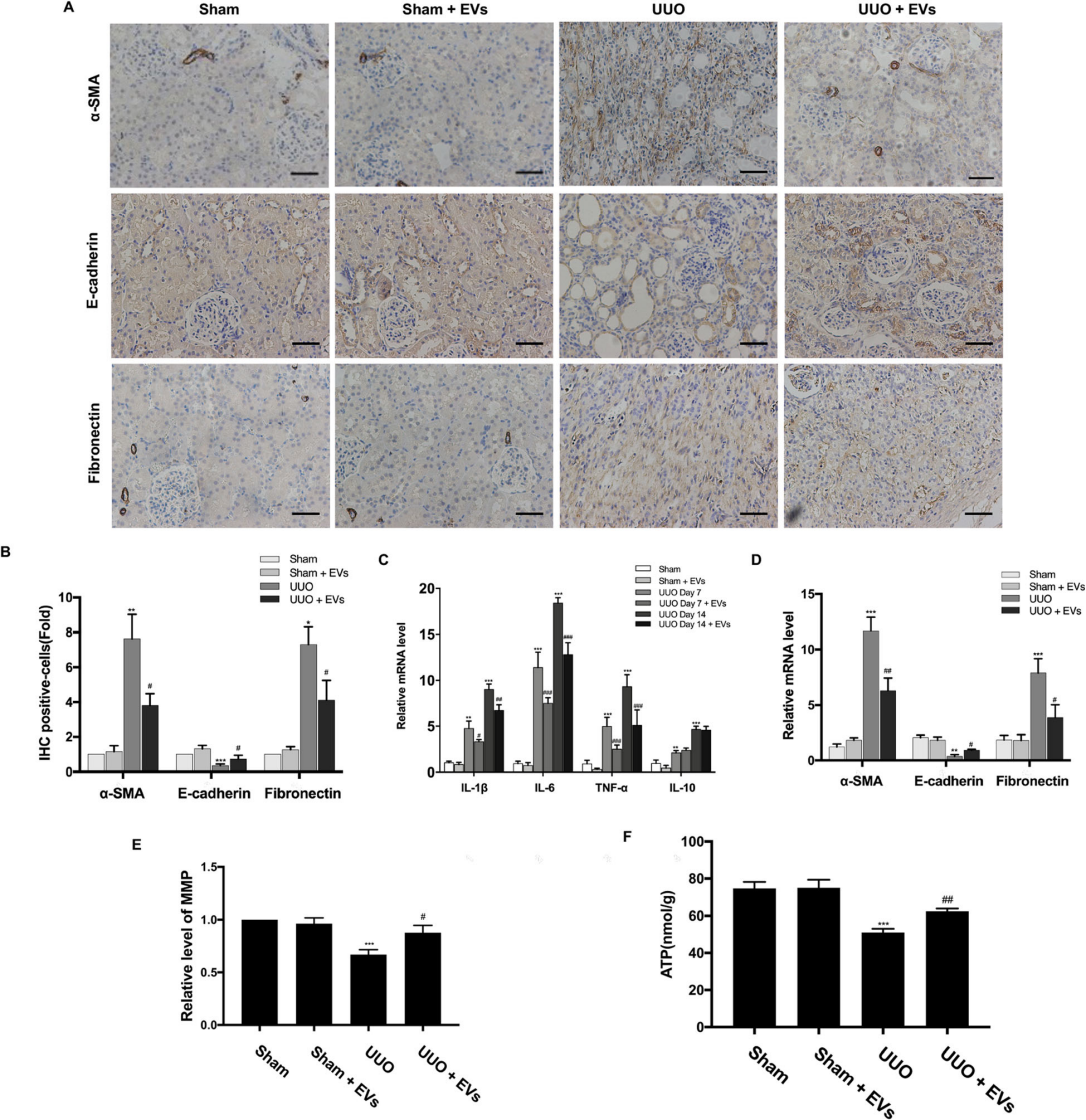
Effects of BMSC-EVs on collagen deposition, fibrosis, inflammation, and mitochondrial dysfunction in UUO kidneys. **a** The locations and expressions of α-SMA, E-cadherin, and Fibronectin were determined by immunohistochemical staining in the kidney sections of rats from different groups. **b** Semi-quantitative immunohistochemical analysis of the EMT-related protein expression in different groups. **c** qRT-PCR analysis of gene expression was performed for inflammatory mediators IL-1β, IL-6, TNF-α, and IL-10 at 7th and 14th day after operation. **d** The mRNA levels of α-SMA, E-cadherin, and Fibronectin were detected by qRT-PCR. **e** Relative level of MMP in different groups. **f** The ATP content of kidneys in different groups. Scale bar 100 μm. *P < 0.05, **P < 0.01, ***P < 0.001 (compared with the Sham group); ^#^*P* < 0.05, ^##^*P* < 0.01, ^###^*P* < 0.001 (compared with the UUO group)

We then assessed pathological changes and collagen deposition by HE and Masson’s trichrome staining at day 14. As shown (Fig. 2e), we observed the infiltration of inflammatory cells and severe structural damage such as tubular dilation, tubular atrophy, and glomerular enlargement in the kidneys of UUO rats. Masson’s trichrome staining revealed severe interstitial collagen deposition in the obstructed kidneys. In the group treated with BMSC-EVs, we observed fewer dilated tubules, less substantially atrophied parenchyma, and reduced collagen deposition compared with untreated rats (Fig. 2f). Immunohistochemical staining showed the same changes in the expression of fibrotic biomarkers (Fig. 3a, b). However, the intravenous administration of BMSC-EVs prevented these changes in fibrotic factor expression in UUO kidneys. Overall, these results indicated that treatment with BMSC-EVs protects against UUO-induced renal pathological changes and fibrosis.

### Treatment with BMSC-EVs protects UUO kidneys against inflammation, mitochondrial dysfunction, oxidative stress, and apoptosis

Progressive inflammation promotes collagen deposition, resulting in TIF in UUO kidneys. Thus, we investigated the effect of BMSC-EV treatment on inflammation in UUO kidneys on days 7 and 14. Quantitative reverse transcription PCR showed that EVs reversed increases of the inflammatory cytokines IL-1β, tumor necrosis factor-α (TNF-α), and IL-6 induced by UUO. Moreover, treatment with BMSC-EVs effectively reduced the elevated expression of the macrophage marker ED-1 in UUO kidneys (Fig. 2g). These results show that treatment with BMSC-EVs reduced inflammation and macrophage infiltration in UUO kidneys. However, unexpectedly, levels of the anti-inflammatory cytokine IL-10 were increased in injured kidneys but did not continue to increase after EV injection. As shown in Fig. 3e, a decline in MMP in the UUO group suggests the occurrence of damage to the mitochondrial membrane; the ATP concentration was also dramatically reduced in the UUO group (Fig. 3f). In contrast, intervention with EVs significantly attenuated mitochondrial damage.

**Fig. 4:**
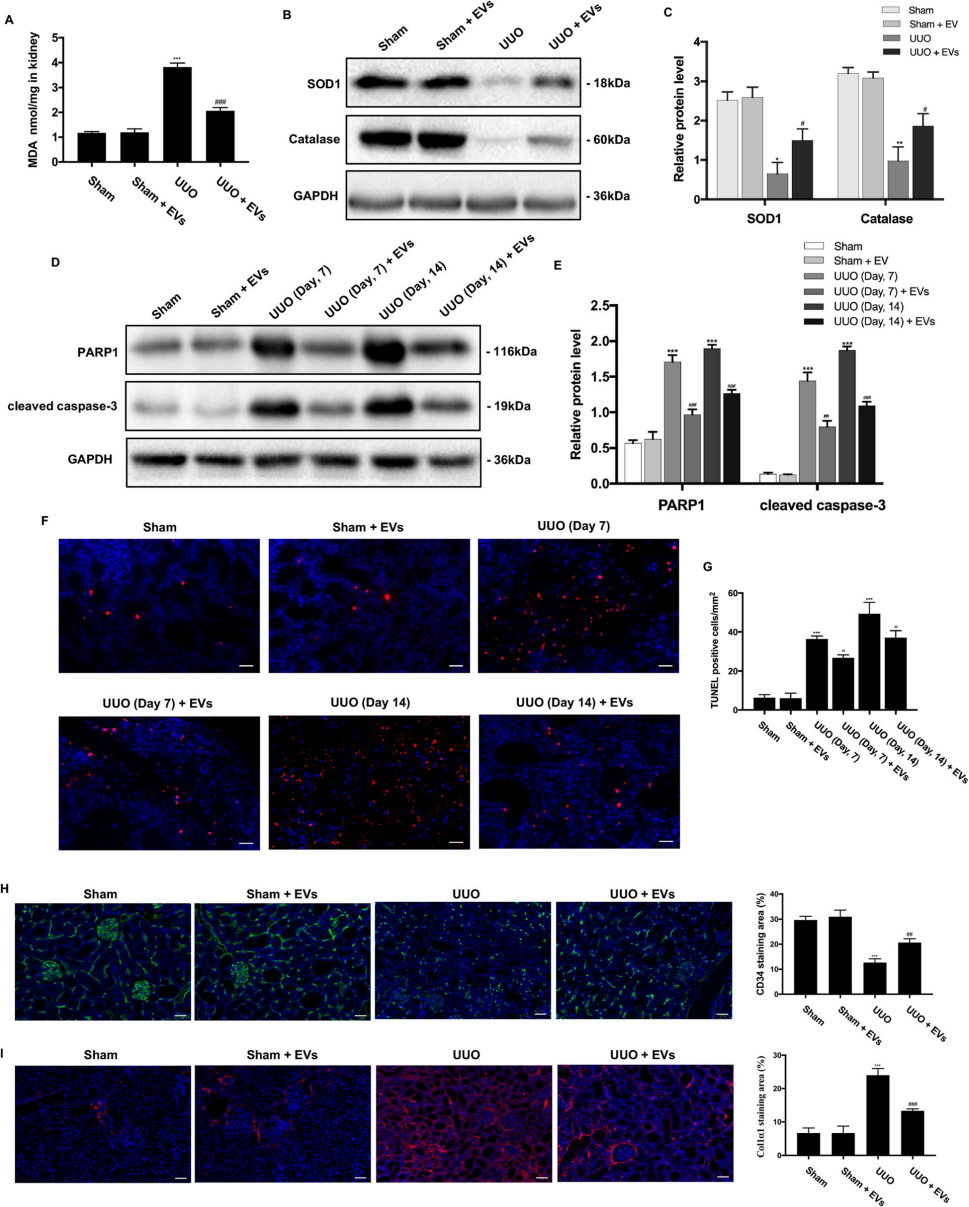
Effects of BMSC-EVs on the oxidative stress, cell apoptosis, renal microvasculature, and peritubular capillary pericytes in UUO kidneys. **a** The MDA content in the kidney was detected by commercial reagent kit. **b** The protein expressions for SOD1 and Catalase were detected by Western blot. **c** The protein levels of SOD1 and Catalase were expressed as arbitrary densitometric units and normalized by the value of GAPDH. **d** The protein expressions for PARP1 and cleaved caspase-3 at 7th day and 14th day after operation were detected by Western blot. **e** The protein levels of PARP1 and cleaved caspase-3 were expressed as arbitrary densitometric units and normalized by the value of GAPDH. **f** Representative images of rat kidneys of different groups by TUNEL staining. **g** TUNEL-positive cells counted in kidneys. **h** IF staining for CD34 revealed the renal microvasculature of kidneys. **i** IF staining for Col1α1 revealed the increase of pericytes in the UUO group. Scale bar 50 μm. *P < 0.05, **P < 0.01, ***P < 0.001 (compared with the Sham group); ^#^*P* < 0.05, ^##^*P* < 0.01, ^###^*P* < 0.001 (compared with the UUO group)

We next investigated the effect of BMSC-EV treatment on oxidative stress-related molecules in UUO kidneys. As shown in Fig. 4a, a significant increase in MDA level in the kidney was observed in the UUO group. Besides, the expression of the antioxidant enzymes SOD1 and Catalase were significantly reduced in UUO kidneys, whereas treatment with BMSC-EVs upregulated these two proteins (Fig. 4b, c). Thus, treatment with BMSC-EVs appeared to diminish oxidative stress in UUO kidneys.

Apoptosis plays a pathogenic role in UUO-induced renal fibrosis. Compared with the Sham group, the expression of the apoptosis markers cleaved caspase-3 and PARP1 was downregulated in rats from the UUO + EVs group on days 7 and 14 (Fig. 4d, e). Furthermore, the number of TUNEL-positive cells in kidney proximal tubules was clearly elevated in the UUO group compared with the Sham group, and this effect was dramatically reduced by the administration of BMSC-EVs (Fig. 4f, g). As expected, renal microvasculature was well-organized in the Sham group. In contrast, the UUO model was characterized by microvascular rarefaction as demonstrated by a reduction in the number of CD34^+^ cells, which were well preserved by BMSC-EVs (Fig. 4h). Additionally, pericyte proliferation was noted after UUO operation with reactivation of Col1α1 expression, a marker of the myofibroblast phenotype (Fig. 4i).

### BMSC-EVs inhibit TGF-β1-induced activation of HK-2 cells

**Fig. 5:**
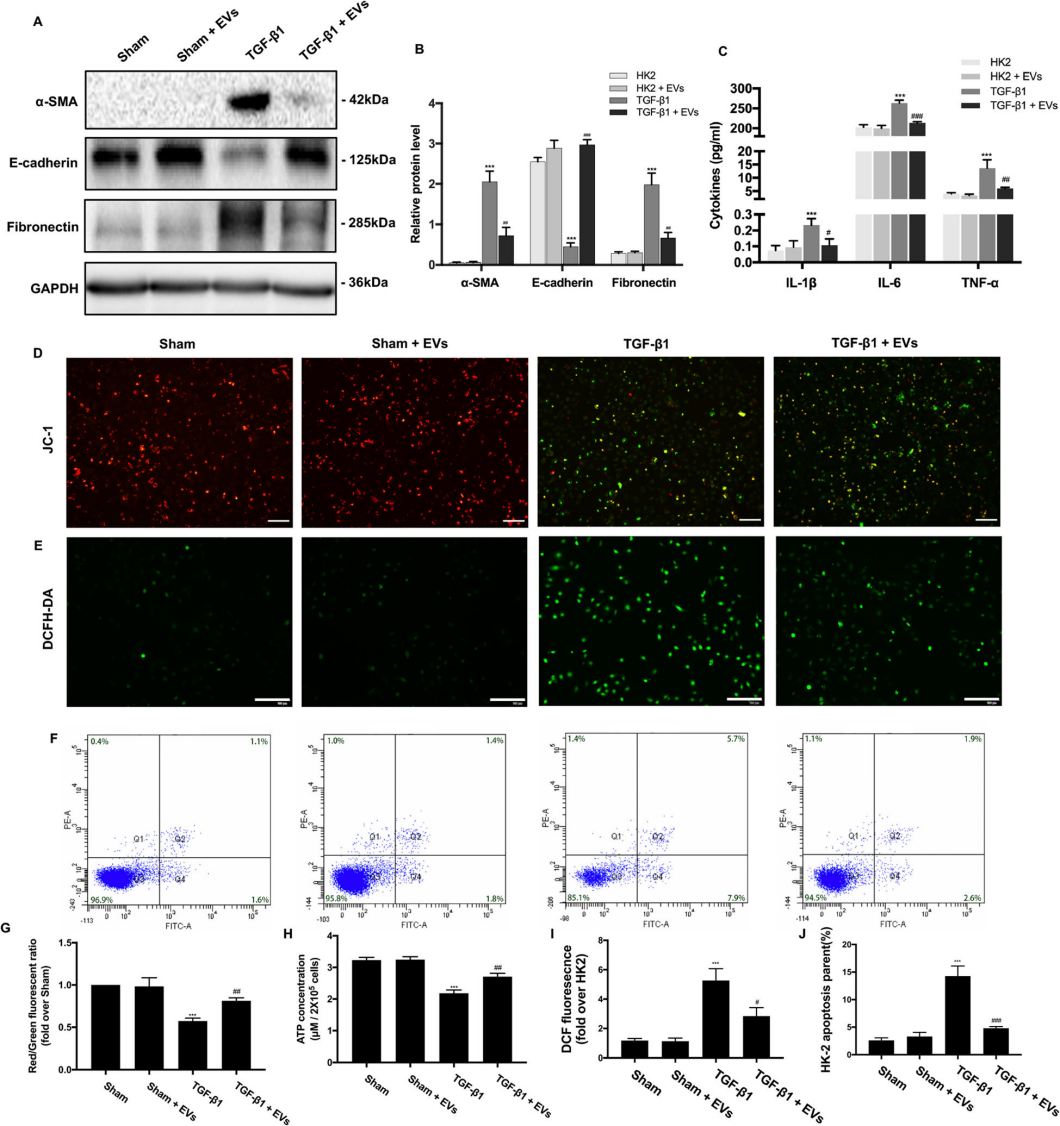
Effects of BMSC-EVs on TGF-β1-induced HK-2 cells. **a** The protein expressions for α-SMA, E-cadherin, and Fibronectin were detected by Western blot. **b** The protein levels of α-SMA, E-cadherin, and Fibronectin were expressed as arbitrary densitometric units and normalized by the value of GAPDH. **c** The levels of IL-1β, IL-6, and TNF-α were detected with ELISA kits. **d**, **g** Representative images of HK-2 cells stained with JC-1 and red/green fluorescent ratio in different groups. **e**, **i** Representative image of HK-2 cells stained with 2′7′-dichlorodihydrofluorescein diacetate (DCFH-DA). Quantification of DCFH-DA. **f**, **j** Rate of cell apoptosis as qualified by flow cytometry. **h** The ATP content of HK-2 cells in different groups. Scale bar 100 μm. *P < 0.05, **P < 0.01, ***P < 0.001 (compared with the Sham group); ^#^*P* < 0.05, ^##^*P* < 0.01, ^###^*P* < 0.001 (compared with the TGF-β1 group)

Next, we treated HK-2 cells with TGF-β1 (10 ng/ml) for 72 h to induce fibrosis and investigated the protective effect of BMSC-EVs against fibrosis, inflammation, mitochondrial dysfunction, oxidative stress, and apoptosis in vitro. To examine the effect of BMSC-EVs on the epithelial–mesenchymal transition (EMT), epithelial markers and mesenchymal markers were assessed by Western blot. We found that treatment with BMSC-EVs triggered a clear decrease in the expression of the mesenchymal markers α-SMA and Fibronectin, while the expression of the epithelial marker E-cadherin was increased compared with HK-2 cells stimulated by TGF-β1 but not when treated with BMSC-EVs (Fig. 5a, b). Moreover, TGF-β1 induced a clear increase in proinflammatory molecule secretion, which was suppressed by treatment with BMSC-EVs (Fig. 5c), supporting our earlier in vivo findings. Not surprisingly, the administration of TGF-β1 induced a decrease of MMP and ATP production, which was blocked by BMSC-EV treatment (Fig. 5d, g, h). Regarding oxidative stress, treatment with BMSC-EVs reduced TGF-β1-induced ROS production in HK-2 cells, as shown in Fig. 5e and i. Consistent with this, apoptosis was significantly reduced in cells treated with BMSC-EVs as assessed by flow cytometry with annexin V-FITC/PI double staining, which was similar to our earlier in vivo findings (Fig. 5f, j).

### Identification of candidate anti-fibrotic factors activated by treatment with BMSC-EVs

**Fig. 6:**
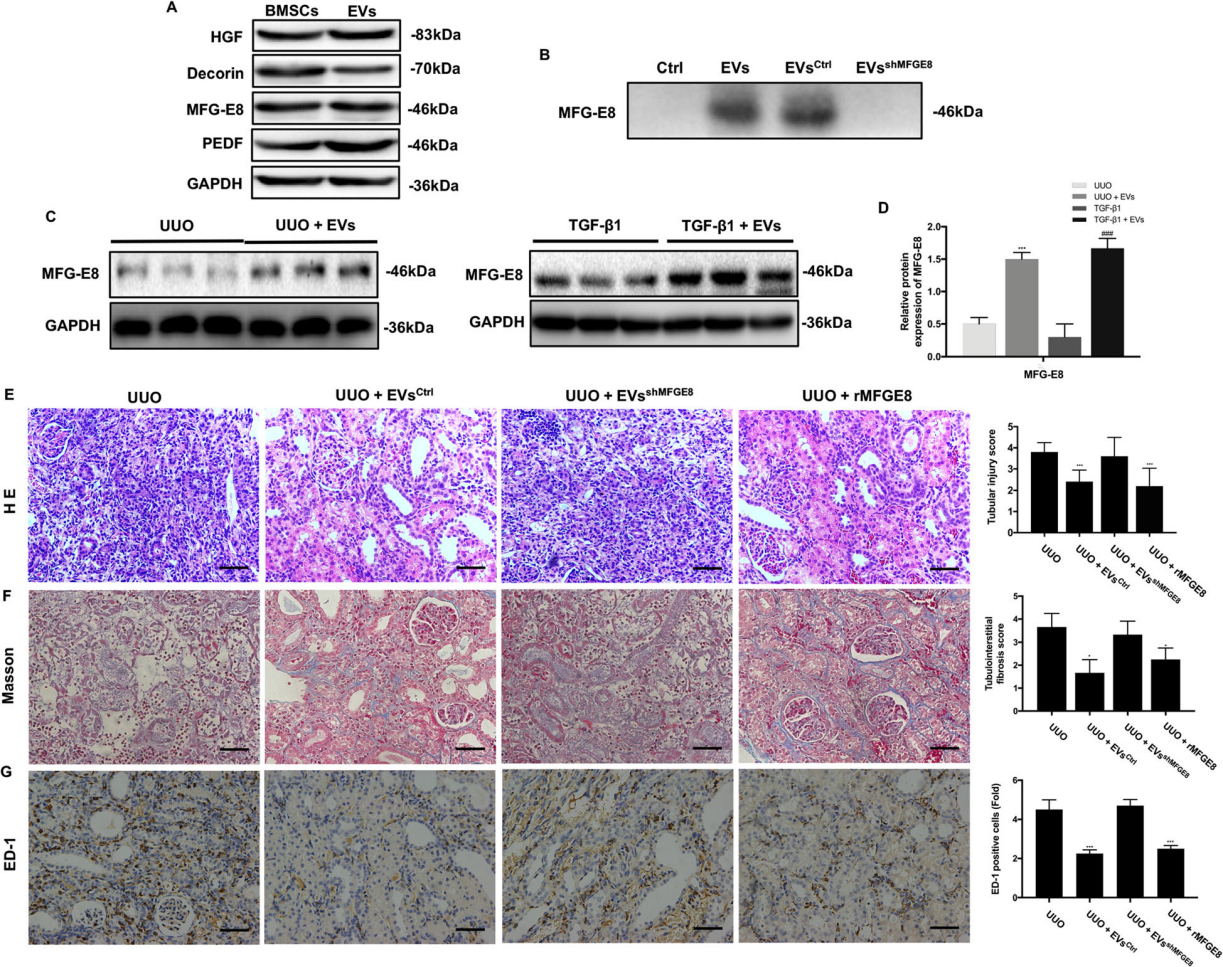
Effect of MFGE8-silenced EVs on tubular damage, collagen deposition, and inflammation in UUO kidneys. **a** The protein expressions of MFG-E8, HGF, PEDF, and Decorin in BMSCs and BMSC-EVs were detected by Western blot. **b** A representative Western blot showing the secretion of MFG-E8 from control, BMSC-EVs, BMSC-EVs^Ctrl^, and BMSC-EVs^shMFGE8^. **c** The MFG-E8 levels in kidneys and HK-2 cells were detected by Western blot. **d** The protein levels of MFG-E8 were expressed as arbitrary densitometric units and normalized by the value of GAPDH. **e** Representative image of the HE staining from kidneys of UUO, UUO + EVs^Ctrl^, and UUO + EVs^shMFGE8^ rats. The bar graph depicts tubular injury scores based on HE staining. Scale bar 100 μm. **f** Representative image of Masson’s trichrome staining from kidneys of UUO, UUO + EVs^Ctrl^, and UUO + EVs^shMFGE8^ rats. The bar graph depicts renal interstitial fibrosis scores based on Masson’s trichrome staining. Scale bar 100 μm. **g** Representative images illustrating the infiltration of ED-1-positive macrophages of rat kidneys in different groups. Quantification of the number of ED-1-positive macrophages pre-field in different groups. Scale bar 150 μm. *P < 0.05, **P < 0.01, ***P < 0.001 (compared with the UUO group); ^#^*P* < 0.05, ^##^*P* < 0.01, ^###^*P* < 0.001 (compared with the TGF-β1 group)

Many proteins delivered by MSCs are anti-fibrotic, including MFG-E8, HGF, PEDF, and **Decorin**. We next examined the expression of these factors in BMSC-EVs by Western blot (Fig. 6a). MFG-E8 has previously been implicated in the MSC-EV-mediated amelioration of liver fibrosis [15], and as expected, it was expressed at high levels in BMSC-EVs. To determine whether MFG-E8 is involved in the response to treatment with BMSC-EVs, we first assessed MFG-E8 expression in BMSC-EVs by Western blot. We also examined MFG-E8 levels in renal tissues and HK-2 cells using Western blot (Fig. 6c). A notable increase in MFG-E8 expression was observed in UUO kidneys treated with BMSC-EVs compared with untreated UUO kidneys, and a similar tendency was observed in TGF-β1-induced HK-2 cells (Fig. 6c, d).

**Fig. 7:**
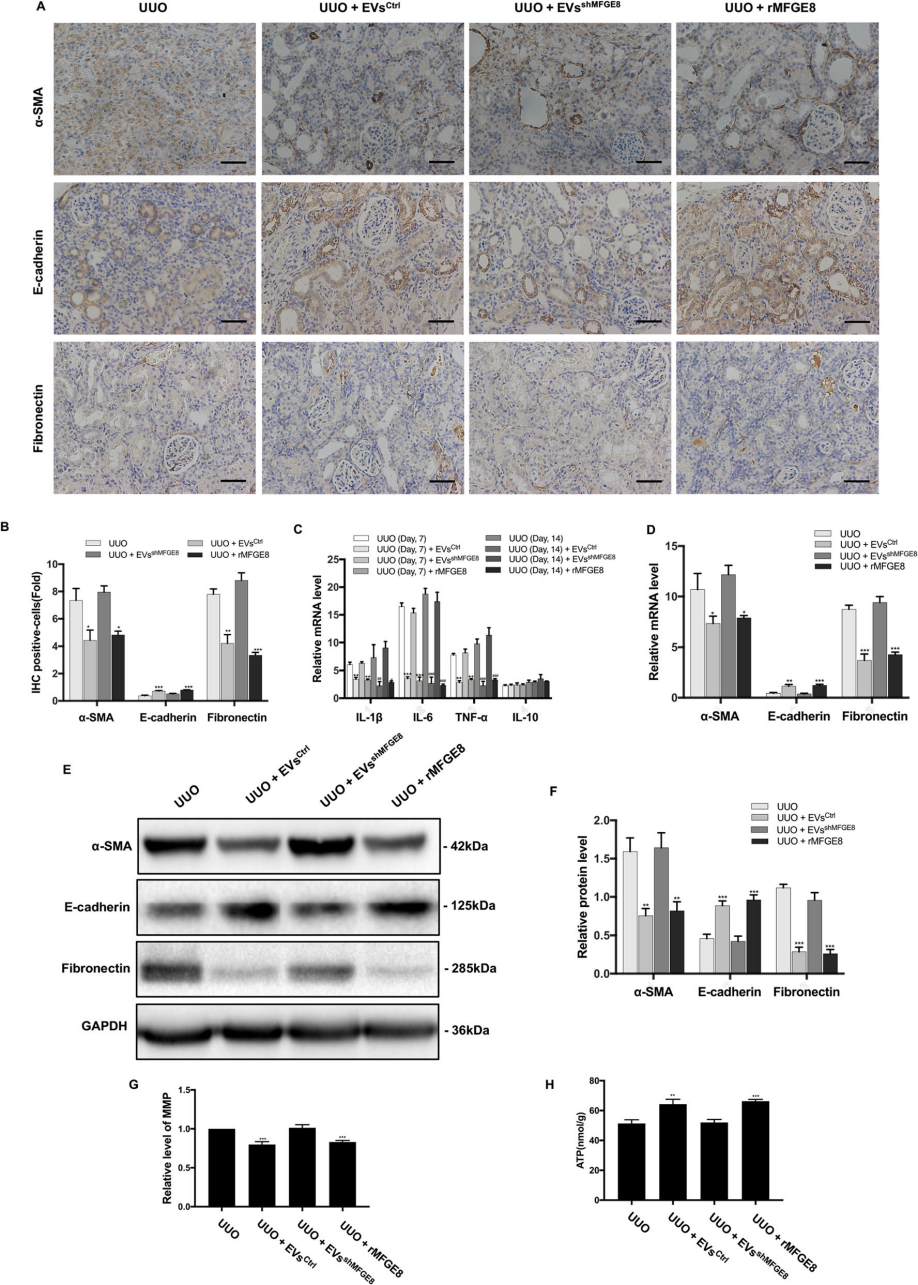
Effects of MFGE8-silenced EVs and rMFGE8 on collagen deposition, fibrosis, inflammation, and mitochondrial dysfunction in UUO kidneys. **a** The locations and expressions of α-SMA, E-cadherin, and Fibronectin were determined by immunohistochemical staining in the kidney sections of rats from different groups. **b** Semi-quantitative immunohistochemical analysis of the EMT-related protein expression in different groups. **c** qRT-PCR analysis of gene expression was performed for inflammatory mediators IL-1β, IL-6, TNF-α, and IL-10 at 7th and 14th day after operation. **d** The mRNA levels of α-SMA, E-cadherin, and Fibronectin were detected by qRT-PCR. **e** The protein expressions for α-SMA, E-cadherin, and Fibronectin were detected by Western blot. **f** The protein levels of α-SMA, E-cadherin, and Fibronectin were expressed as arbitrary densitometric units and normalized by the value of GAPDH. **g** Relative level of MMP in different groups. **h** The ATP content of kidneys in different groups. Scale bar 100 μm. *P < 0.05, **P < 0.01, ***P < 0.001 (compared with the UUO group)

Next, BMSCs were transfected with lentiviral vectors expressing MFG-E8 small hairpin RNAs to silence MFG-E8 expression, which was verified by Western blot (Fig. 6b). Examination of the effect of MFGE8-silenced BMSC-EVs in renal TIF induced by UUO revealed more severe renal damage compared with rats treated with normal BMSC-EVs (Fig. 6e). Masson’s trichrome staining also showed increased extracellular matrix deposition after treatment with MFGE8-silenced BMSC-EVs compared with normal BMSC-EVs (Fig. 6f). As shown in Fig. 7d–f, the expression of fibrosis markers in rats injected with MFGE8-silenced BMSC-EVs was not significantly altered compared with UUO kidneys. Immunohistochemical staining revealed similar findings (Fig. 7a, b). Additionally, inflammatory cytokine mRNA levels and macrophage infiltration were not obviously changed (Figs. 6g and 7c), and there was no difference in MMP or ATP content between the UUO groups with and without MFGE8-silenced BMSC-EV treatment (Fig. 7g, h).

**Fig. 8:**
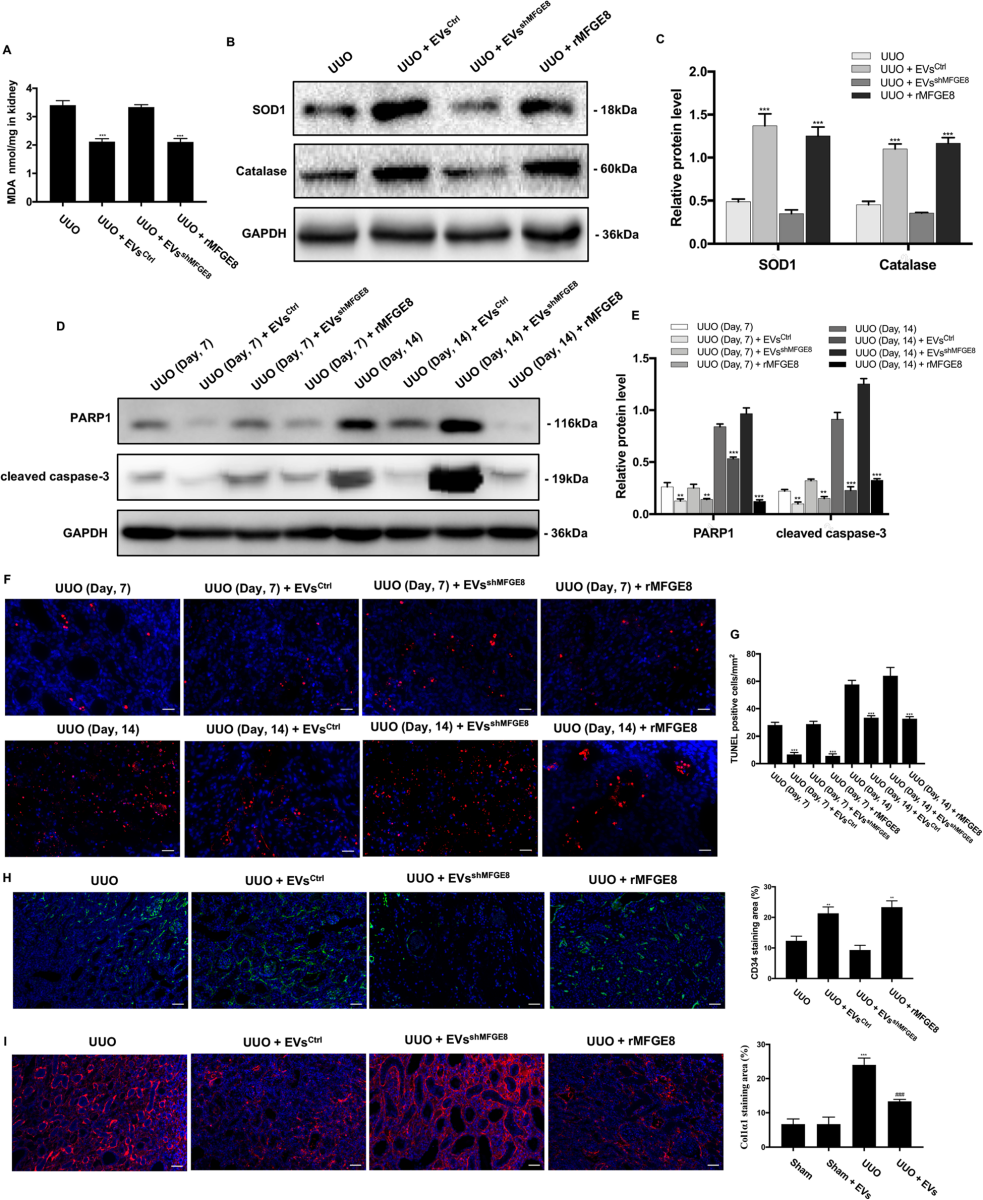
Effects of MFGE8-silenced EVs and rMFGE8 on the oxidative stress, cell apoptosis, renal blood flow, renal function, renal microvasculature, and peritubular capillary pericytes in UUO kidneys. **a** The MDA content in the kidney was detected by commercial reagent kit. **b** The protein expressions for SOD1 and Catalase were detected by Western blot. **c** The protein levels of SOD1 and Catalase were expressed as arbitrary densitometric units and normalized by the value of GAPDH. **d** The protein expressions for PARP1 and cleaved caspase-3 at 7th day and 14th day after operation were detected by Western blot. **e** The protein levels of PARP1 and cleaved caspase-3 were expressed as arbitrary densitometric units and normalized by the value of GAPDH. **f** Representative images of rat kidneys of different groups by TUNEL staining. **g** TUNEL-positive cells counted in kidneys. **h** IF staining for CD34 revealed the renal microvasculature of kidneys. **i** IF staining for Col1α1 revealed the increase of pericytes in the UUO group. Scale bar 50 μm. *P < 0.05, **P < 0.01, ***P < 0.001 (compared with the UUO group)

**Fig. 9:**
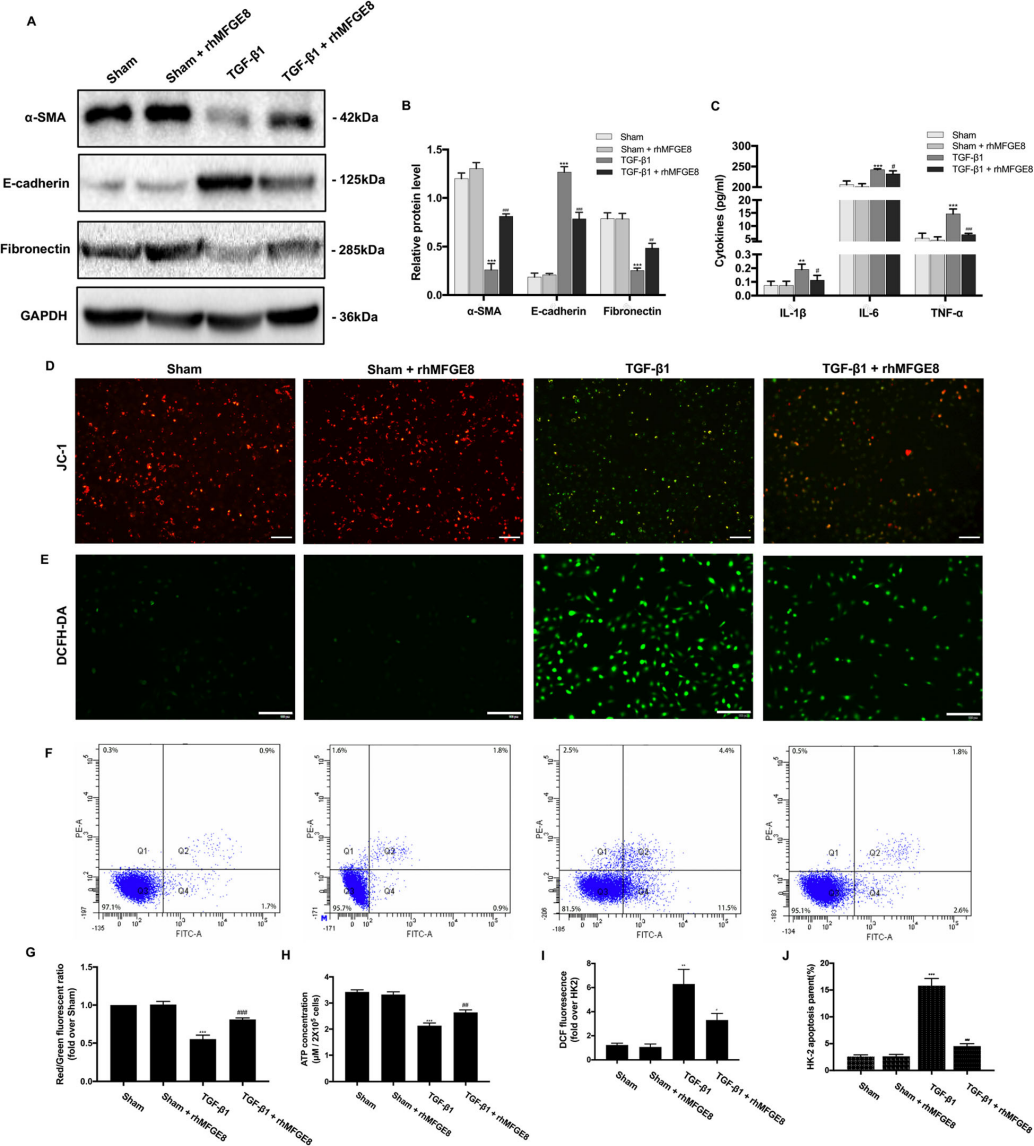
Effects of rhMFGE8 on TGF-β1-induced HK-2 cells. **a** The protein expressions for α-SMA, E-cadherin, and Fibronectin were detected by Western blot. **b** The protein levels of α-SMA, E-cadherin, and Fibronectin were expressed as arbitrary densitometric units and normalized by the value of GAPDH. **c** The levels of IL-1β, IL-6, and TNF-α were detected with ELISA kits. **d**, **g** Representative images of HK-2 cells stained with JC-1 and red/green fluorescent ratio in different groups. **e**, **i** Representative image of HK-2 cells stained with 2′7′-dichlorodihydrofluorescein diacetate (DCFH-DA). Quantification of DCFH-DA. **f**, **j** Rate of cell apoptosis as qualified by flow cytometry. **h** The ATP content of HK-2 cells in different groups. Scale bar 100 μm. *P < 0.05, **P < 0.01, ***P < 0.001 (compared with the Sham group); ^#^*P* < 0.05, ^##^*P* < 0.01, ^###^*P* < 0.001 (compared with the TGF-β1 group)

We then evaluated the expression of oxidative stress-related proteins. As shown in Fig. 8a, the MDA level was higher in the group treated with MFGE8-silenced BMSC-EVs compared with normal BMSC-EVs. The expression levels of SOD1 and Catalase were lower in the group treated with MFGE8-silenced BMSC-EVs compared with normal BMSC-EVs (Fig. 8b, c), and a greater degree of apoptosis was seen (Fig. 8d–g). Rats in the UUO + EV^shMFGE8^ group showed no difference in microvasculature and pericyte compared with those in the UUO group (Fig. 8h, i). However, as expected, recombinant human MFG-E8 reversed renal injury seen after the UUO operation (Fig. 8) and in TGF-β1-induced HK-2 cells (Fig. 9). Taken together, these results suggest that MFGE8-silenced BMSC-EVs provide little protection against the adverse renal effects induced by UUO.

### MFG-E8 expressed by BMSC-EVs attenuates fibrosis in HK-2 cells by inhibiting RhoA/ROCK signaling

**Fig. 10:**
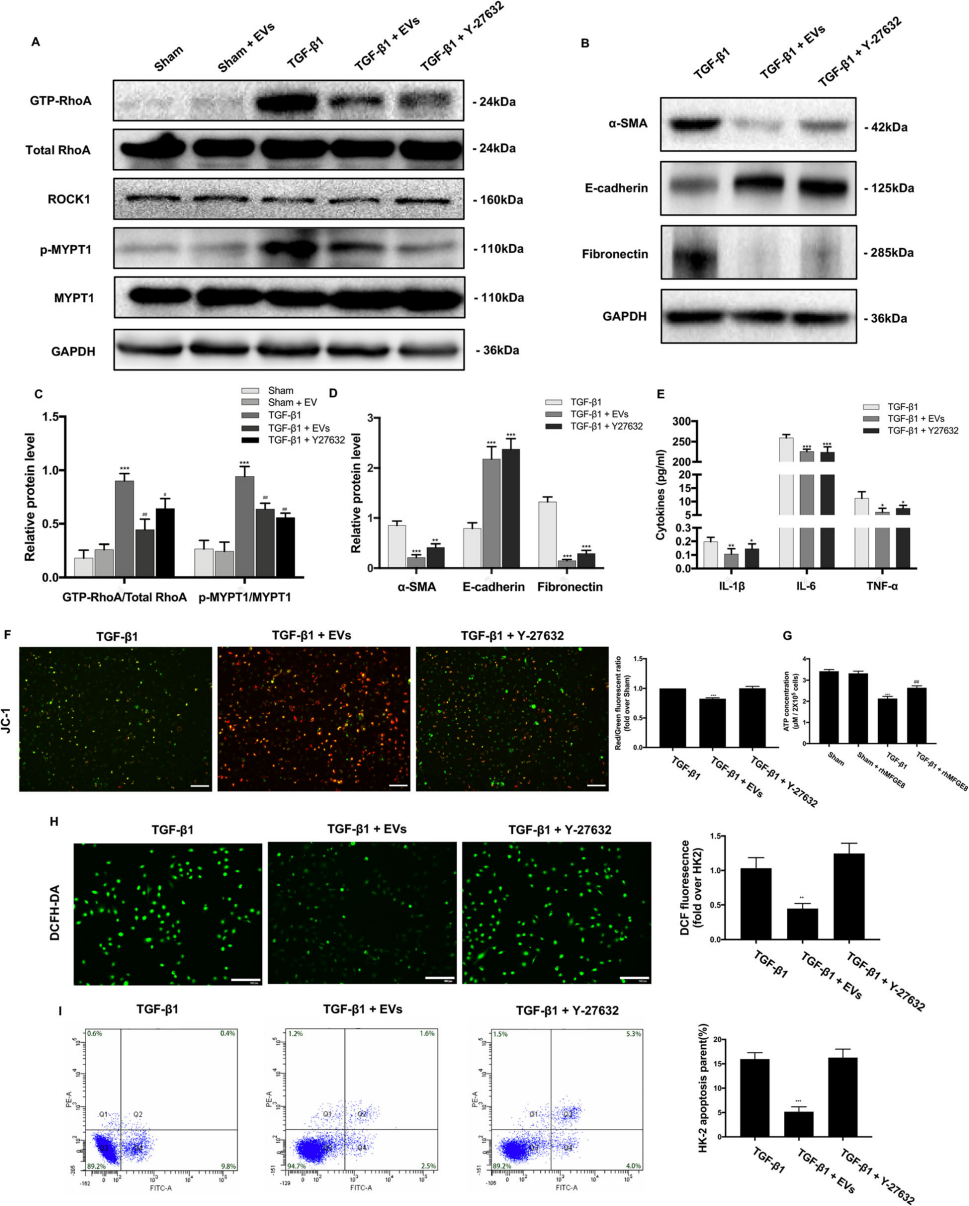
BMSC-EVs deliver nephroprotection via partly inhibiting the RhoA/ROCK pathway. **a**, **c** The protein expressions for GTP-RhoA, total RhoA, ROCK1, p-MYPT1, and MYPT1 were detected by Western blot. **b**, **d** The protein expressions for α-SMA, E-cadherin, and Fibronectin were detected by Western blot, and the protein levels of α-SMA, E-cadherin, and Fibronectin were expressed as arbitrary densitometric units and normalized by the value of GAPDH. **e** The levels of IL-1β, IL-6, and TNF-α were detected with ELISA kits. **f** Representative images of HK-2 cells stained with JC-1 and red/green fluorescent ratio in different groups. **g** The ATP content of HK-2 cells in different groups. **h** Representative image of HK-2 cells stained with 2′7′-dichlorodihydrofluorescein diacetate (DCFH-DA). Quantification of DCFH-DA. Scale bar 100 μm. **i** Rate of cell apoptosis as qualified by flow cytometry. *P < 0.05, **P < 0.01, ***P < 0.001 (compared with the Sham group); ^#^*P* < 0.05, ^##^*P* < 0.01, ^###^*P* < 0.001 (compared with the TGF-β1 group)

We next determined whether RhoA/ROCK signaling is involved in the TGF-β1-induced activation of HK-2 cells. We performed a RhoA activity assay for RhoA activation and used Western blot to determine the expression of MYPT1, a major effector of ROCK1 and an indicator of ROCK1 activity. The level of activated GTP-bound RhoA was dramatically increased after co-culturing with TGF-β1, whereas treatment with BMSC-EVs attenuated TGF-β1-mediated RhoA activation (Fig. 10a). Additionally, there was an approximately 3.5-fold increase in p-MYPT1 levels in the TGF-β1 group compared with unstimulated HK-2 cells. However, treatment with BMSC-EVs attenuated the increase in p-MYPT1 levels (Fig. 10a).

Then, we treated cells with the ROCK inhibitor Y-27632 for further verification. Compared with the TGF-β1 group, the group treated with Y-27632 exhibited higher E-cadherin expression levels and lower α-SMA and Fibronectin levels, similar to the effect seen with BMSC-EVs (Fig. 10b, d). Furthermore, the administration of Y-27632 significantly reversed changes in inflammatory cytokine expression levels (Fig. 10e). However, there was no change in TGF-β1-induced mitochondrial dysfunction, oxidative stress, or renal cell apoptosis (Fig. 10f–i). These results indicate that the RhoA/ROCK pathway is involved in fibrosis and inflammation but not mitochondrial dysfunction, oxidative stress, or cell apoptosis in TGF-β1-induced HK-2 cells.

## Discussion

Renal fibrosis develops in response to a host of insults such as growth factors and stress molecules, and the development goes through several important phases including the activation of renal interstitial fibroblasts, excessive and progressive accumulation of extracellular matrix, EMT, inflammatory cell infiltration, and tubular cell apoptosis [21–23]. In this study, we found that BMSC-EVs can reduce renal fibrosis by producing MFG-E8, which inhibits the RhoA/ROCK pathway.

Because of the severe consequences of renal fibrosis, an increasing number of studies have focused on developing effective treatments [19, 24, 25]. UUO and TGF-β1 induction of HK-2 cells are well-established in vivo and in vitro models [26, 27], respectively. We adopted these models to test the therapeutic effect and mechanisms of BMSC-EVs. Emerging data supports that BMSCs exert their positive effect by releasing EVs, which may be more effective. EVs, which are typically approximately 30–1000 nm in size and are composes of exosomes, microvesicles and apoptotic bodies, are membranous bodies that are released by almost all cells [28, 29]. EVs attract more attention in the field of organ regeneration because they selectively packed with s proteins, mRNAs, microRNAs, and lipids [30]. More researches support the view that EVs have important physiological effect in disease progression. This process may represent an alternative approach to stem cell therapy that could help attenuate disease progression [8].

Inflammation plays a vital role in the initiation and progression of renal injury in chronic renal fibrosis [31, 32], which eventually leads to the destruction of renal parenchyma. Previous studies have shown that damaged tubular epithelial cells recruit inflammatory cells to the renal interstitial compartment, resulting in the production of a myriad of proinflammatory and pro-fibrotic cytokines by the infiltrated inflammatory cells [33]. We found that treatment with BMSC-EVs reverse UUO-induced increase of inflammatory cytokines TNF-α, IL-6, and IL-1β and macrophage infiltration, indicating that EVs effectively protect against UUO-induced renal fibrosis by inhibiting the inflammatory response. However, the expression of anti-inflammatory cytokine IL-10 was not clearly increased by treatment with BMSC-EVs, which may reflect the differential effect of BMSC-EVs in preventing inflammation.

Mitochondria as a vital organelle take part in energy production and maintain tissue homeostasis [34, 35]. Abnormal function in mitochondria was relative to loss of ATP synthesis and increase of ROS production. Similar to the dramatic decrease in ATP content, we found that UUO significantly decreased MMP as a component of the overall proton motive force and that treatment with BMSC-EVs prevented the loss, as expected.

Elevated oxidative stress is another pivotal cause of renal fibrosis [36], which is an imbalance between the oxidation system and the endogenous antioxidant defense system. Our findings confirmed these results, with in vivo data showing that UUO largely increased the content of MDA. Besides, the activities of SOD1 and Catalase were suppressed in the UUO group and that intravenous BMSC-EV injection partly prevented enzyme inactivation. Our in vitro results were also consistent with these findings.

Apoptosis, a key pathological process in renal fibrosis, can be triggered by either extrinsic or intrinsic pathways [37–39]. If apoptotic cells are not quickly cleared, secondary necrosis will occur, which can lead to further damage [40]. We observed a reduction in apoptosis in the group receiving BMSC-EV injection. Previous studies have shown that SOD1 and Catalase reduce the expression of pro-apoptotic molecules [41], suggesting that BMSC-EVs suppress oxidation-induced apoptosis by inhibiting the intrinsic apoptotic pathway.

Pericytes are an important source of interstitial myofibroblasts [42]. Col1α1+ cells are located beneath endothelial cells, juxtaposed with CD34-expressing endothelial cells. After UUO, we found that Col1α1 expression rises, which indicates that peripheral cells gradually turn into myofibroblasts after migrating into the matrix, resulting in matrix deposition. Regarding glomerular filtration rate (GFR), which regularly declined in the obstructed kidney but significantly increased in the contralateral kidney after UUO, suggesting an early functional compensation in the UUO model [43]. In irreversible UUO, only GFR value of contralateral kidney can be measured, indicating that the value cannot reflect to injury severity. The reversible UUO model is used to research renal recovery following relief of obstruction, which is similar to a transiently obstructing ureteral stone in patients. Renal function parameters such as GFR and renal blood function (RBF) can accurately reflect the difference between the bilateral kidney and the recovery of the obstructed kidney in reversible UUO [44]. Study with a reversible UUO may be conducted in the future.

The levels of beneficial and harmful factors contained in the EVs may result in different therapeutic outcomes [15]. Therefore, identifying beneficial compounds is critical for developing more efficient therapies. MFG-E8 has generated a great deal of interest because of its potential to attenuate fibrosis [15]. Our investigation confirmed the effect of MFGE8 within BMSC-EVs, and the nephroprotective effects of EVs virtually disappeared following transfection with the lentivirus unexpectedly. These findings show that MFG-E8 is a key EV molecule that contributes to fibrotic regression.

Our findings also suggest a mechanistic basis for how BMSC-EVs exert their nephroprotective effects. Many studies have illustrated the importance of RhoA/ROCK signaling in regulating fibrosis [45, 46], so we hypothesized that the RhoA/ROCK pathway may contribute to BMSC-EV-mediated nephroprotection. Consistent with our expectations, we found that BMSC-EVs downregulated RhoA/ROCK expression in vitro, and RhoA/ROCK inhibitor primarily reversed fibrosis progression.

There were three limitations to our study. First, in irreversible UUO, only renal function parameters of the contralateral kidney, such as GFR and renal blood function (RBF), can be measured, indicating that the value cannot reflect to injury severity, which are different from the model of reversible UUO [44]. The follow-up time points of 2 weeks in this study may not be long enough for the final analysis. Study with a longer evaluation period may be needed in the future. Second, while our study showed that BMSC-EVs reduce renal fibrosis in part by inhibiting RhoA/ROCK, further research is needed to determine how BMSC-EVs exert antioxidant and antiapoptotic activities in the context of renal fibrosis. Third, characterization of EV’s cargo is of significantly important because the cargo can provide information to EV’s biogenesis, targeting, and cellular function, which may be a source of information for disease prognosis and response to treatment. Therefore, a broader analysis of the cargo is needed in our further studies.

## Conclusions

In summary, our findings show that EVs derived from BMSCs can attenuate renal fibrosis. More importantly, we present a novel mechanism by which BMSC-EVs deliver MFG-E8 to renal cells and inhibit inflammation, oxidative stress, apoptosis, and fibrosis, in part by downregulating the RhoA/ROCK pathway. This study highlights the potential therapeutic usefulness of BMSC-EVs in clinical applications.

## Data Availability

All data generated or analyzed during this study are included in this published article.

## Abbreviations

BCA: Bicinchoninic acid
CKD: Chronic kidney disease
BMSCs: Bone marrow mesenchymal stem cells
ECL: Enhanced chemiluminescence
ECM: Extracellular matrix
EMT: Epithelial–mesenchymal transition
EVs: Extracellular vesicles
GFR: Glomerular filtration rate
HE: Hematoxylin and eosin
HK-2: Human renal proximal tubular epithelial
IHC: Immunohistochemical
MFG-E8: Milk fat globule–EGF factor-8
PBS: Phosphate-buffered saline
RBF: Renal blood function
SD: Sprague-Dawley
SPF: Specific pathogen free
TEM: Transmission electron microscopy
TGF-β1: Transforming growth factor-β1
TIF: Tubulointerstitial fibrosis
TNF-α: Tumor necrosis factor-α
UUO: Unilateral ureteral obstruction
